# From theoretical concepts to policies and applied programmes: the landscape of integration of oral health in primary care

**DOI:** 10.1186/s12903-018-0484-8

**Published:** 2018-02-15

**Authors:** Hermina Harnagea, Lise Lamothe, Yves Couturier, Shahrokh Esfandiari, René Voyer, Anne Charbonneau, Elham Emami

**Affiliations:** 1 School of Public Health, Public Health Research Institute, Université de Montréal , Montréal, Québec H3N 1X7 Canada; 2 School of Social Work, Université de Sherbrooke, Sherbrooke, Québec J1H 4C4 Canada; 3 Faculty of Dentistry, McGill University , Montreal, Quebec H3C 3J7 Canada; 4 Faculty of Dentistry, Université de Montréal, Montréal, Québec H3T 1J4 Canada; 5 CRCHUM, Université de Montréal, Montreal, Quebec Canada

**Keywords:** Oral health, Integration, Primary care

## Abstract

**Background:**

Despite its importance, the integration of oral health into primary care is still an emerging practice in the field of health care services. This scoping review aims to map the literature and provide a summary on the conceptual frameworks, policies and programs related to this concept.

**Methods:**

Using the Levac et al. six-stage framework, we performed a systematic search of electronic databases, organizational websites and grey literature from 1978 to April 2016. All relevant original publications with a focus on the integration of oral health into primary care were retrieved. Content analyses were performed to synthesize the results.

**Results:**

From a total of 1619 citations, 67 publications were included in the review. Two conceptual frameworks were identified. Policies regarding oral heath integration into primary care were mostly oriented toward common risk factors approach and care coordination processes. In general, oral health integrated care programs were designed in the public health sector and based on partnerships with various private and public health organizations, governmental bodies and academic institutions. These programmes used various strategies to empower oral health integrated care, including building interdisciplinary networks, training non-dental care providers, oral health champion modelling, enabling care linkages and care coordinated process, as well as the use of e-health technologies. The majority of studies on the programs outcomes were descriptive in nature without reporting long-term outcomes.

**Conclusions:**

This scoping review provided a comprehensive overview on the concept of integration of oral health in primary care. The findings identified major gaps in reported programs outcomes mainly because of the lack of related research. However, the results could be considered as a first step in the development of health care policies that support collaborative practices and patient-centred care in the field of primary care sector.

## Background

Primary health care has been defined by the World Health Organization (WHO) *as essential health care based on practical, scientifically sound and socially acceptable methods and technology made universally accessible to individuals and families in the community through their full participation and at a cost that the community and country can afford to maintain […]. It is the first level of contact of individuals, the family and community with the national health system bringing health care as close as possible to where people live and work, and constitutes the first element of a continuing health care process* [1].

Primary health care includes a large range of services such as oral health care and encompasses a variety of health care providers across the public, private and non-government sectors. The integration of oral health into primary care has been implemented in some health care systems to reduce the burden of oral health disease and to improve access to oral health care, especially for disadvantaged people and communities [2]. This approach empowers health promotion and oral disease prevention, and favours health equity. It includes various domains such as risk assessment, oral health evaluation, preventive intervention, communication and education as well as interprofessional collaborative practice [3].

Despite the growing attention being generated towards integrated oral health care and the support of medical organizations such as the American Academy of Family Physicians for an oral health framework [4], the documentation on oral health integrated models is disparate, and it remains unclear how and in what contexts this approach is being applied and is successful in practice.

Therefore, as presented in the published protocol [5], a comprehensive scoping review has been conducted by our research team, to answer several research questions on the concept of the primary oral health care approach. This article presents the findings in regard to the following research questions:

- What are the main conceptual and applied models as well as policies that exist on the integration of oral health in primary care?
- To what extent the integration of oral health in primary care improve oral health outcomes, especially for vulnerable and disadvantaged populations?

## Methods

The review was conducted using the six-stage methodological framework of Levac et al. [6] including 1) identifying the research question, 2) searching for the relevant studies, 3) selecting studies, 4) charting and collating the data, 5) summarizing and reporting the results, and 6) consultation with stakeholders to inform the review.

Since the protocol of this scoping review has been published previously [5], only a brief summary is presented here. Using specific mesh terms and keywords, a detailed search strategy was designed with the help of an expert librarian at Université de Montréal. Various data bases including OVID NCBI, EBSCOhost, ProQuest, Databases in Public Health, Databases of the National Institutes of Health (health management and health technology), Health Services and Sciences Research Resources, Health Services Research & Health Care Technology, Health Services Research Information Central, Health Services Research Information Portal, Health Services Technology Assessment Texts, and Healthy People 2020 have been searched. The snowballing technique was used to identify additional relevant resources and the grey literature. Publications in English or French from 1978 to April 2016 have been reviewed. All research studies irrespective of study design in which the integration of oral health into primary care is the primary focus of the publication were included. Commentaries, editorials and individual points of view were excluded.

Two calibrated reviewers (HH, EE) have independently reviewed the title and abstract according to defined eligibility criteria (kappa = 0.83). After the complete review of selected publications, data were extracted and charted by the same reviewers (HH, EE). At each step, reviewers’ disagreements were discussed with other research team members and resolved by consensus. This paper presents specifically the results on the policies, applied programs and outcomes. The findings in regard to barriers and facilitators were presented in a previous publication [7].

### Summarizing and reporting the results

**Fig. 1:**
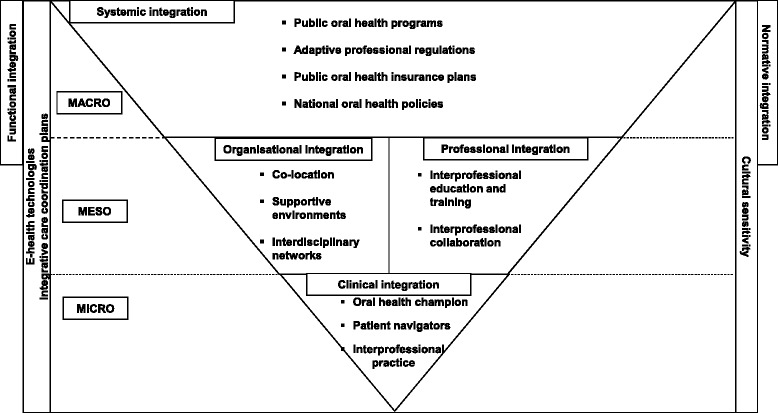
Strategies for integration of oral health into primary care based on the Valentijn et al. integration framework

A content analysis was used to synthesize, summarize and report the study’s findings. This included a descriptive analysis of the results and a schematization based on the integration framework (Rainbow model), proposed by Valentijn et al. [8] (Fig. 1). The data were also classified into two tables (Tables 1 and 2), according to the oral health outcomes and type of programs. Extracted data were grouped into various categories as shown in Tables. A triangulation was conducted by the scoping review team and the results were discussed and revised.

**Table 1:** Integration of oral health into primary care: Summary of integrated oral health care programmes

Authors, Country/ Year	Program type/Target population	Program main strategy	Oral health care provider	Main outcomes
Bain & Goldthorpe, Canada/1972	University-initiated outreach /Aboriginal community	• Assigned full-time dentists to community’s hospital, providing dental services in nursing stations and satellites	Dentists & dental residents	• Creation of supportive environment • Demonstration of feasibility, replicability
Rozier et al., USA/2003	Statewide community clinics preventive program/Low income children 0–3 years old	• Reimbursement of non-dental care providers for preventive dental services	Paediatricians, family physicians, nurses and other health care professionals in community clinics	• ↑ trained medical professionals (88% participation rate) • Wide geographical oral health coverage • ↑ 2.8 times the number of practices with submitted claims over one-year period • ↑ follow-up visits
Wysen et al., USA/ 2004	Public-health based program /Low-income children ≤5 years old	• Empowering case management model • Co-location of dental and medical clinics • Providers cross-training • Community education and outreach	Case managers, community agency staff, physicians, public health nurses, dentists and dental hygienists	• Successful training of community care providers • ↑ numbers of screening, dental visits and oral health services • 109% ↑ in fluoride varnish applications over 10-month period
Heuer, S., USA/2007	School-linked clinics /Low income children	• Contractual partnership with a local community dental health center and employment of dental hygienists at school • Training of school nurse practitioners for screening of oral diseases	Nurse practitioners and dental hygienists	• ↑ Parents’ satisfaction • ↓ of no-show rates for dental care
Stevens et al., USA/2007	Oral health-oriented prenatal practice /Pregnant low income adolescents	• Incorporation of evidence-based oral health guidelines in prenatal care • Inclusion of dental consultations in prenatal sessions	Nurse midwives and nurse practitioners, paediatric dental consultant, obstetrician, physician, social worker and nutritional specialist	• ↑ Patients’ satisfaction
Dugdill, L. & Pine, CM., UK/ 2011 Pine CM & Dugdill L, UK/2011	Global multi-objective public-health programs in collaboration with National Dental Associations, the member associations of Federal Dental International (FDI) and Unilever Oral Care/Wide-range population groups	• Public-private partnership • Training of day care workers to deliver oral health promotion in day care centers (Philippines) • Education of future parents (Poland) • Training of dental educators (Indonesia) • Training for dentists (Nigeria)	Non-dental care providers Dentists	• Raised awareness of oral health in 1 million people from 36 countries • ↑ capacity building to deliver oral health in 36 countries • Improvement of oral health status in children over a ten-year period
Brownlee, B., USA/2012 Nycz, G., USA/ 2014 Maxey, H., USA/2015 Taflinger et al., USA/2016 Acharya, A., USA/2016 Gesko, DS., USA/2016	Patient-centered dental homes targeting various models of care: physician led model, administration-driven model, culture of integration, interprofessional collaboration, dental outreach coordinator/Low income children, pregnant women and diabetic patients	• Co-location of dental and medical care • Oral health champion modelling to provide oral health care in the primary care setting • Implementation of protocol for referral protocols • Cross-training of dentists and medical providers	Primary health care providers & clinical assistants Dental care team (dentist, dental hygienist, dental assistant, dental therapist)	• ↓ oral health risk factors for some of the models including • ↑ number of patients receiving dental care in all delivery models • Implementation of systematic and reproducible risk assessment tool for periodontal disease and oral cancer • Some programs based on physician-led models were not sustainable
Ramos-Gomez, FJ., USA/2014	University initiated program in partnership with community-based organizations	• Training of all staff involved • 3-month rotation for dental paediatric residents	Non-dental providers and dental residents	• 672 patients and 1500 visits over a 3 year period • More than 42% of the children had 2 or more visits • 138 patients were maintained caries-free and the programme prevented lesions from progressing in 51 patients
Leavitt Partners, USA/2015	Dental services integrated in accountable care organizations/ Public & private-insured population groups	• Co-location of medical and dental care • Case management • Higher reimbursement rates for care coordination via medical providers • Reimbursement of non-dental and dental care providers for preventive dental services • Contracting with dental associations to provide dental care in private and public settings • Empowering dental leadership	Dentists, care coordinators, non-dental care providers, outreach and referral team	• ↓ 55% of operating room utilization for children’s dental care under sedation • ↓ 50% of dental pain complaints • ↓ 9.1% in emergency visits over one-year period • ↑ 3.3% outpatient visits over one- year period
Wooley, S., Australia/ 2016	Community-controlled primary health care service /Aboriginal population	• Care coordination to enable two-way referrals and information exchange between staff and community	Dentist and dental consultant, nurses	• Fissure sealants and fluoride varnish to 100% of the children over a five- year period • ↓ emergency attendance rates over a five- year period • DMFT = 0 in 53.1% of 12 years old children and dmft = 0 in 16.9% of 0–4 year old children over a five-year period

**Table 2:** Integration of oral health into primary care: Summary of oral health outcomes

Author, Year/Country	Study objective/Study design	Setting/Target health care users	Data collection	Indicators	Main outcomes
Haughney et al.*,* 1998/UK	To develop and evaluate a model of integrated medical and dental care/Cohort study	Co-located medical and dental practices under National Health System regulation/General population	• Postal questionnaire • Health records archive	• Number of registered joint patients • Information discrepancies • Joint work practices • Number of secondary referrals	• 90% increase in the number of registered joint patients over a 3-year period • ↓ discrepancies • 42% ↑ in 0–5 year olds’ number of joint visits • 24% ↑ in > 75 year olds’ number of joint visits • ↓ need for secondary referrals (*n* = 41) over a 3-year period
Pronych et al., 2010/USA	To examine the efficacy of systems approach and training nursing staff on the oral health of nursing home residents/Pilot study	Long-term care facilities (LTC)/Geriatric population	• Clinical examination • Interviews with the oral health coordinators	• Simplified debris index (DI-S) of residents at baseline, 2, 6 and 12 month follow-up • Success and barriers of the model • Feedback on the oral health coordinator’s role	• Statistically significant improvement in the oral hygiene of LTC residents
Dyson et al., 2012/ Australia	To examine the cost-effectiveness of a rural and remote networked hub-and-spokes model / Retrospective economic analysis	Fixed dental services embedded in Aboriginal Health Services/Aboriginal communities	• Services activities data	• Cost-to-value ratio	• Cost-to-value ratio average: 1.61 • Not statistically significant difference between sites, according to the Accessibility/Remoteness Index of Australia
Gerritsen et al., 2013/ Netherlands	To compare the cost and effects of integrated care versus incidental care/Observational study	Long-term care facilities/Geriatric population	• Clinical examination • Administrative data	• Oral health status • Cost of dental care	• Integrated care ↓ dental treatment needs • Integrated care ↑ cost and time spent on dental care
Hom et al., 2013/USA	To evaluate the adherence to early and periodic screening, diagnosis and treatment guidelines for medical practices/ Observational study	Medical practices/Medicaid registered children	• Medicaid administrative data base	• Number of states adhering to the best oral health practices • Number of states requiring dental referral by age 1	• 88% of states adhered to the content and timing of best oral health practices • 33% of the states adhered to the best oral health practice by requiring referral by age 1
Kranz et al., 2014/USA	To examine the association between the type of the service provider (primary care provider/PCP, dentist) and subsequent dental-caries related treatment (CRT) and CRT payment/Retrospective study	North Carolina Medicaid / Children aged 3–5 years	• Medicaid enrollment and claim files from 2000 to 2006	• CRT • CRT payment	• Statistically significant difference among children visiting PCPs, dentist or both in regard to CRT and CRT payments • The dentist provider type was associated with ↑ CRT and ↑ CRT payments per year
Langelier et al., 2015/USA Langelier, M., 2015/USA	To identify effective approaches to integrating primary care and oral health services delivery /Case studies	Federally qualified health care centers across United States /Vulnerable population groups	• Interviews and focus group discussion	• Number of dental clinics • Number of dental personnel • Number of dental visits • Attendance • Referral mechanisms • Number of primary care providers trained	• ↑ number of dental clinics • ↑ number in dental residents • After 3 months, some clinics were fully booked, with 3–4 week waits for appointments • Electronic health record interoperability
Grisanti et al., 2015/USA	To examine the performance of Federally Qualified Health Centers over 5-year period (2007–2012)/ Observational study	Community health center’s dental department /Medicaid, uninsured and privately insured patients	• Administrative records	• Oral health age-specific indicators: number of dental visits, number of received oral health services/year, number of received preventive interventions/year, percentage of preventive measures, number/percentage of preventive visits	• 87% ↑ in the total number of patients who received at least one dental visit over 5-year period • About 50% ↑ in the total number of patients who received preventive interventions • 27% ↑ comprehensive exam • 97% ↑ in number of patients having preventive interventions • No increase in Medicaid patients having a dental procedure • 56% ↑ in restorative procedures for 65 + • 140% ↑ in preventive services
DiMarco et al., 2016/USA	To test the feasibility of integrating primary preventive interventions into the practice of nurses, registered dieticians and students	Sites of the Supplemental Nutrition Program for women, children and infants/Low income preschool children	• Dental screening and administrative records • Parent/Guardian oral health survey	• Number of preventive fluoride varnishes and education sessions • Oral force diversity, capacity and flexibility • Interprofessional collaboration	• Fluoride varnish applied to 40% of children in order to reduce the number of cavities by 25% • Enhanced education of 40% of women and mothers at both sites • Establishing a dental home for 75% of children • Expanded the scope of practice of RD, RN, NP • Enhanced cross training opportunities

### Stakeholder consultations

The stakeholders included representatives of academic health care organizations, policy decision-makers and health care professionals, as well as community and patient representatives. They were engaged through a workshop [9] and several meetings in the various steps of the scoping review including preliminary reviews of a few published articles, discussions on the study research questions and study findings, and developing effective dissemination strategies.

## Results

### Description of included studies

**Fig. 2:**
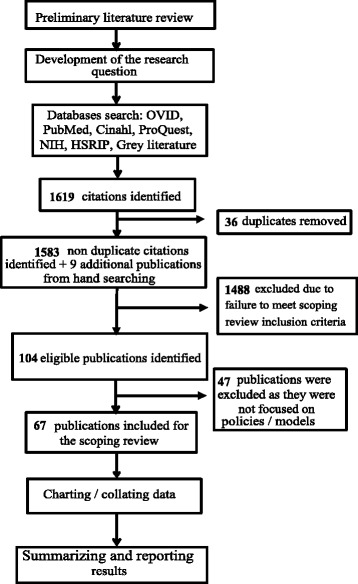
Flowchart of the scoping review

As presented in Fig. 2, from the total of 104 publications included in the full review, 67 reported on theoretical and applied models as well as policies in regard to the integration of oral health into primary care. These publications were from 6 countries: the United States of America (USA), Australia, Canada, Brazil, the Netherlands and the United Kingdom (UK). The publications included conceptual frameworks (*n* = 6), policies and strategic plans (*n* = 35), programmes descriptions (*n* = 16) and related applied research studies (*n* = 10). The majority of research studies were published in the last decade and were conducted in the USA.

### Conceptual frameworks

The review identified two frameworks developed in the USA. The first, used in the Integration of Oral Health and Primary Care Practice (IOHPCP) Initiative**,** was proposed by the Health Resources and Services Administration [3, 10]. It covers five domains including risk assessment, oral health evaluation, preventive intervention, communication and education and inter-professional collaborative practices [3, 11]. This framework has been adopted by some Federally Qualified Healthcare Centers (FQHC) for the implementation of their tailored programmes [12]. The second, the Oral Health Delivery Framework, was recently developed in partnership with primary care and dental care clinicians, policy makers and stakeholders from medical, dental, and nursing associations, as well as end-users. The activities proposed in this framework promote proactive coordination between dental and non-dental primary care providers and include: screening patients for oral diseases, identifying particularly high-risk populations, offering fluoride varnish for paediatric patients and high-risk adults, performing patient education, dietary counseling and oral hygiene training, and developing structured documentation and referral processes [13]. Since 2015, this framework has been implemented in 19 primary health care organizations from public services and private practice in five US states, to examine its feasibility and sustainability [14].

### Policies and strategic plans in the integration of oral health in primary care

The scoping review found that policies and strategic plans in regard to integration of oral health in primary care are rooted in universal acceptance of the primary health care approach established by the WHO Alma-Ata declaration of 1978 [15]. The first strategic plan was published in 1982 through collaboration of organizations such as the World Dental Federation (FDI) and the WHO [16]. The scoping review identified an important WHO strategic plan entitled “Oral health: Action plan for promotion and integrated disease prevention,” which was presented to the World Health Assembly and the Executive Board in 2007 [17]. Accordingly, the 193 WHO member countries agreed on implementation of the WHO Global Oral Health Programme [17–19]. In the last decade, all of these countries have set general policies for oral health action, using these guidelines with the concept of common risk factor [18–21]. However, because of the worldwide variation in oral health care organizational systems [22], the specific policies on oral health integrated care differ not only between countries, but also within countries at national and provincial levels. In most countries, policies have focused on care coordination plans, rather than fully oral health integrated strategies. In some countries such as Canada, Australia, the USA and the UK, policies for integrated care put emphasis on specific population groups such as early childhood, children and adults with special needs, elders, rural and remote communities, as well as Aboriginal and Indigenous populations [23–34].

As a pioneer in the category of developing countries and based on its health reform entitled Unified Health System, Brazil has implemented different governmental, federal and municipal policies to completely integrate oral health professionals into multidisciplinary primary care teams [35, 36]. In 2004, Brazil launched a National Policy for Oral Health (NPOH) and linked this specific strategic plan to other public health care policies including the reorganization of primary care, the reorganization of specialized care, fluoridation of the water supply and oral health surveillance [37]. According to the reviewed publications, these policies were successful in the implementation of a sustainable integrated oral health care network [37] and optimizing oral health care services for all groups of citizens, despite some persistent challenges such as cost and resistance from the private oral health care sector [38, 39].

In a number of industrialized countries, various strategies have been tailored to foster WHO principles. In 2011, 42 US states adopted policies under Medicare/Medicaid to encourage general physicians, paediatricians and other primary care providers to provide some basic oral health care [40–43]. The US Health Department of Health and Human Services oral health strategic framework 2014–2017 recommended the primary oral health care approach as a means to overcome the segmentation of health care delivery [10, 44].

In Europe, a collaboration of the Association for Dental Education in Europe, the Council of European Chief Dental Officers, the European Association of Dental Public Health and the European Dental Health Foundation permitted the implementation of a platform to support oral health policies through the development of cross-sectoral approaches and collaboration with health and social care professions [45].

In the UK, integrated dental care services are governed by the National Dental Health Services board [32, 46]. Following the Health and Social Care Act adopted in 2012, dental professionals collaborate with various stakeholders and Local Authority Public Health teams to achieve continuity of care across private and public sectors [32, 47]. The British Dental Association supports strategic collaborations with all health and social care professionals to enable global comprehensive care and to reduce oral health inequalities [48].

### Integrated oral health care programmes and related applied research outcomes

Tables 1 and 2 summarizes the identified integrated oral health care programmes and related applied research outcomes [12, 49–73]. In general, programmes designed to integrate oral health into primary care have been implanted in the public sector and in partnership with various private and public health organizations, governmental bodies, schools and universities (Table 1). These programmes use various strategies to empower integrated care, including building interdisciplinary networks, training non-dental care providers, oral health champion modelling, enabling care linkages and care coordinated process, as well as the use of e-health technologies [57–71].

In many countries a ‘high-risk strategy’ approach has been used to create integrated oral health care programmes. Accordingly, integrated care has focused on groups with the highest risk factor levels for oral diseases such as: child and maternal age-groups, the elderly and persons with physical and mental disabilities, low-income population groups, as well as rural, remote, Indigenous communities.

As an example, about 80% of the FQHC in the USA, which provide health care services to underserved populations, also deliver either comprehensive or preventive dental services [74]. According to the technical report conducted by Maxey et al. [65] and as presented in Table 1, five FQHCs have been identified as leaders in integrating oral health with primary care.

Our scoping review also identified some programmes initiated by dental schools in collaboration with community-based organizations. Thus, the *Infant Oral Care Program* was developed by the University of California (UCLA) to increase family-centred access to care and utilization of dental services [69]. Another example is the Rochester Adolescent Maternity Program, created in 2003, which provides oral health educational guidelines within prenatal services [61]. In 1969, the First Nations and Inuit Health branch of Health Canada, in collaboration with the University of Toronto, developed the “Sioux Lookout Project” [57]. Through this programme, dental clinics and facilities were integrated in the nursing stations in remote First Nations Communities. Dental services were offered by fly-in dentists and certified dental assistants. Similarly, the Centre for Rural and Remote Oral Health in Australia uses networked “hub and spoke models of care” in which dental clinics are integrated in rural and remote Aboriginal Medical Centres [52].

Although this scoping review didn’t identify major publications on sustained outcomes and cost/benefit analysis for these programmes, most of the publications mentioned that integrated primary health care services increase performance of the health care system by creating a supportive environments [12, 49, 53, 57–59, 62, 63, 65, 69–73]. Although supportive environments can potentially improve oral health outcomes, from the review it was not clear to what extent these outcomes were improved, and if the needs of vulnerable and disadvantaged populations were completely addressed (Table 2). Positive outcomes included enhanced interprofessional collaboration, satisfaction of non-dental primary care providers with the oral health care training, increased performance of non-dental care providers, and patient satisfaction with care delivery [50–52, 54, 56, 58–61, 64, 66–69]. Objective outcomes included an increase in the number of trained non-dental health care providers, the number of dental visits, screening and preventive acts, referrals, as well as a decrease in the percentage of patients’ dental treatment non-attendance and finally improved access to dental care [12, 49–52, 54, 55, 60, 69, 70, 72, 73]. Haughney et al. (1998) reported on the follow-up of a co-located model of integrated care and demonstrated that after three years, the total number of medical/dental visits as well as dental visits by the 0–5 year age group and by the elderly increased by 90%, 32% and 24%, respectively [49]. Gerritsen et al. [51]compared integrated versus incidental dental care in two nursing homes in a city in the Netherlands with 35,000 inhabitants. In the centre providing integrated care, although the cost of dental care increased by 86%, the average dental care time for patients was 20 times greater and translated into a reduction of more than 40% of dental treatment needs among seniors [51].

## Discussion

This scoping review maps the literature to identify and describe models and policies of the integration of oral health in primary care. Almost all the publications reviewed in this study highlighted the need for effective policies on interdisciplinary approaches to improve the oral health of disadvantaged population groups.

The review confirmed that *primary oral health care* is not a new concept and in fact is the foundation of the United Nations Millennium Declaration Goal statement: *“All people, everywhere, shall have access to a skilled, motivated and facilitated health worker within a robust health system.”* [75].

However, theoretical and conceptual models in oral health are not well developed in comparison to medical fields. In fact, the various taxonomies of integrated care are still not well differentiated and applied in the field of oral health. In a working document, the WHO has provided an overview of taxonomies and models of integrated care [76]. According to this document six types of integration have been introduced by Lewis et al.: organizational, functional, service, clinical, normative and systemic [77]. This taxonomy has been used to analyze the level and the mechanism of integration. Furthermore, integration has also been classified into horizontal and vertical integration, which could occur in a real or virtual manner, as described by Curry and Ham [78]. The breadth, level and intensity of integration varied from integration health care models for specific groups to whole population [79], from micro and meso to macro level, as well as from partial to full integration. These taxonomies were used in the development and implementation of various integrated care models, including three main models: individual models of integrated care, group- and disease-specific models, as well as population-based models [76].

It seems that in the field of dental care, mostly individual and group-specific models of integrated care have been promoted in order to coordinate the care for high-risk and vulnerable patients and to empower the continuity and delivery of dental care. Although in countries such as Canada, integrated care delivery models like PRISMA (Program of Research to Integrate the Services for the Maintenance of Autonomy) were developed more than a decade ago, oral health has not been included due to the fact that dental care is covered mostly by the private sector. However, in some countries such as Brazil, health policy makers have implemented policies that favour population-based models such as “Smiling Brazil”, the Brazilian National Oral Health Policy (PNSB). These models recognize that oral health is linked to environmental and societal factors and needs to be improved within supportive environments and by using culturally appropriate strategies. The main limitation of population-based models is their considerable cost, which often makes their implementation difficult or unlikely [80]. Several Brazilian publications [81, 82] show that despite the effectiveness of PNSB in terms of oral health care and access, implementation of the principles of the policy continue to encounter difficulties in some cities even 10 years after its adoption.

This is why combined models seem to be both privileged and realistic, given that the expenses encountered by health care organizations are the main influence in the majority of identified models for oral health integration into primary care.

On the other hand, the choice of integrated oral health care models in different countries depends on the clinical services organization, vision and values in the community, governance systems and policies [7]. For example, in the USA and Australia, strategies for oral health integration into primary care are oriented towards oral health clinical competencies achievement for non-dental primary care providers, whereas in some other countries, the policy of professional associations is to protect the population and the profession of dentistry based on academic qualifications, which may not respond to the need of disadvantaged populations.

This scoping review has some limitations that should be noted. First, as with all scoping reviews, it was restricted to the selected French and English publications. Second, since the majority of identified publications provided descriptions of demonstration projects or programmes without describing the outcomes, it was often difficult to assess the degree and breadth of integration of a programme, or obtain a complete understanding of the programme’s impact. Finally, scoping reviews, as opposed to systematic reviews, do not critically appraise individual studies and the risk of bias [6, 83].

## Conclusions

This work highlighted the importance of policies promoting the integration of oral health in primary care and the implementation of interdisciplinary public health programs to improve the oral health of disadvantaged population groups.

Scientific, evidence-based and rigorous evaluation research are needed to provide data on cost-effectiveness and sustained outcomes of oral health integrated models. These researches will encourage health care systems toward implementation of oral health care policies and programs in various countries and populations.

## Abbreviations

FDI: World dental federation
FQHC: Federally qualified health care centers
IOHPCP: Integration of Oral Health and Primary Care Practice
NPOH: National Policy for Oral Health
PNSB: Brazilian National Oral Health Policy
PRISMA: Program of Research to Integrate the Services for the Maintenance of Autonomy
UCLA: University of California
UK: United Kingdom
USA: United States of America
WHO: World health organization

## References

[1] World Health Organisation (WHO). Health Systems Strengthening Glossary. World Health Organisation. 2017. http://www.who.int/healthsystems/hss_glossary/en/index8.html. Accessed 12.04 2017.

[2] Petersen PE (2014). Strengthening Of oral health systems: oral health through primary health care. Med Princ Pract.

[3] U.S. Department of Health and Human Services. Integration of Oral Health and Primary Care Practice. Health Resources and Services Administration. 2014. https://www.hrsa.gov/publichealth/clinical/oralhealth/primarycare/integrationoforalhealth.pdf. Accessed 3 Apr 2017.

[4] American Academy of Family Physicians. Oral health. American Academy of Family Physicians. 2017. http://www.aafp.org/patient-care/public-health/oral-health.html. Accessed 5.03 2017.

[5] Emami E, Harnagea H, Girard F, Charbonneau A, Voyer R, Bedos CP, Chartier M, Wooton J, Couturier Y. Integration of oral health into primary care: a scoping review protocol. BMJ Open. 2016;6(10) 10.1136/bmjopen-2016-013807. 10.1136/bmjopen-2016-013807 PMC5073498 27798039

[6] Levac D, Colquhoun H, O’Brien KK. Scoping Studies: advancing the methodology. Implement Sci. 2010;5(69) 10.1186/1748-5908-5-69 PMC2954944 20854677

[7] Harnagea H, Couturier Y, Shrivastava R, Girard F, Lamothe L, Bedos CP, Emami E. Barriers and facilitators in the integration of oral health into primary care: a scoping review. BMJ Open. 2017; 10.1136/bmjopen-2017-016078. 10.1136/bmjopen-2017-016078 PMC5623507 28951405

[8] Valentijn P, Schepman SM, Opheij W, Bruijnzeel, MA. Understanding integrated care: a comprehensive conceptual framework based on the integrative functions of primary care. Int J Integr Care. 2013(Jan–Mar). 10.5334/ijic.886 PMC3653278 23687482

[9] Emami E, Couturier Y, Girard F, Torrie J (2016). Integration of oral health into primary health Care Organization in Cree Communities: a workshop summary. J Can Dent Assoc.

[10] U.S. Department of Health and Human Services. Oral health strategic framework 2014–2017. Public health reports (Washington, DC : 1974). 2014;131(2). https://www.hrsa.gov/sites/default/files/oralhealth/oralhealthframework.pdf. Accessed 6 Feb 2018. PMC4765973 26957659

[11] U.S. Department of Health and Human Services. Considerations for Oral Health Integration in Primary Care Practice for Children. Rockville, Maryland: U.S. Department of Health and Human Services. 2012. https://www.hrsa.gov/oralhealth/oralhealthprimarychildren.pdf. Accessed 7.04 2017.

[12] Langelier M, Moore J, Baker BK, Mertz E. Case Studies Of 8 federally Qualified health centers: strategies to integrate oral health with Primary Care 2015. http://www.oralhealthworkforce.org/wp-content/uploads/2015/11/FQHC-Case-Studies-2015.pdf. Accessed 6.02.2018

[13] Hummel J, Phillips KE, Holt B, Hayes C. Oral Health - An Essential Component of Primary Care-White Paper Seattle, WA: Qualis Health.. 2015. https://dphhs.mt.gov/Portals/85/publichealth/documents/OralHealth/White-Paper-Oral-Health-Primary-Care.pdf. Accessed 1.07 2017.

[14] Qualis Health. Qualis health publishes comprehensive guide for implementing oral health. Integration. 2015; http://www.safetynetmedicalhome.org/sites/default/files/Guide-Oral-Health-Integration.pdf. Accessed 1.07 2017

[15] World Health Organisation (WHO). Declaration of Alma Ata. International conference on primary health care: World Health Organisation.1978.

[16] World Dental Federation (FDI) (1982). Global goals for oral health in the year 2000. Int Dent J.

[17] Petersen PE (2008). World Health Organization global policy for improvement of oral health – world health assembly 2007. Int Dent Journal.

[18] Petersen P, Bourgeois D, Bratthall D, Ogawa H. Oral Health information systems--towards measuring progress in oral health promotion and disease prevention. Bull World Health Organ. 2005;83(9) PMC2626332 16211160

[19] Petersen PE, Kwan S (2004). Evaluation Of community-based oral health promotion and oral disease prevention--WHO recommendations for improved evidence in public health practice. Community Dent Health.

[20] World Health Organisation. Proposed 10-year regional plan on oral health. World Health Organisation. 2006. http://apps.who.int/iris/handle/10665/168660. Accessed 1.07 2017.

[21] New South Wales Government. Oral Health 2020: A Strategic Framework for Dental Health in NSW. 2013. http://www.health.nsw.gov.au/oralhealth/Pages/oral_health_2020.aspx. Accessed 28.06 2017.

[22] Widström E, Eaton KA (2004). Oral Healthcare systems in the extended European union. Oral Health Prev Dent.

[23] Federal Provincial and Territorial Dental Directors. A canadian oral health strategy. 2005. http://individual.utoronto.ca/accessandcare/Patterson.pdf. Accessed 30.03 2015.

[24] Rowan-Legg A (2014). Oral Health care for children – a call for action. Paediatr Child Health.

[25] Canadian Academy of Health Science. Améliorer l’accès aux soins de santé bucco-dentaire pour les personnes vulnérables vivant au Canada. 2014. http://www.msss.gouv.qc.ca/professionnels/documents/sante-dentaire/Archives_2015/Paul_Allison.pdf. Accessed 01.07 2017.

[26] Dwyer J, O’Donnell K, Lavoie J, Marlina U, Sullivan P. The overburden report: contracting for Indigenous health services. Darwin: Cooperative Research Centre for Aboriginal Health. 2009. https://www.healthinfonet.ecu.edu.au/key-resources/bibliography/?lid=16708. Accessed 1.07 2017.

[27] Williams S, Jamieson L, MacRae A, Gray C. Review of indigenous oral health. Australian Indigenous HealthInfoNet. 2011. http://www.healthinfonet.ecu.edu.au/other-health-conditions/oral/reviews/our-review. Accessed 1.07 2017.

[28] Tripartite First Nations Health Plan. Healthy Smiles for Life – BC’s First Nations and Aborigianl Oral health Strategy. 2014. http://www.fnha.ca/about/news-and-events/news/healthy-smiles-for-life-bcs-first-nations-and-aboriginal-oral-health-strategy.

[29] Abrams M, Chung L, Fisher M, Lugtu K, Rose S, Stookey J. San Francisco children's oral health strategic plan 2014–2017. 2014. http://assets.thehcn.net/content/sites/sanfrancisco/Final_document_Nov_2014_20141126111021.pdf. Accessed 1.07 2017.

[30] American Academy of Paediatrics (2008). Preventive oral health intervention for pediatricians. Paediatrics.

[31] British Society for Disability and Oral Health. Clinical Guidelines and Integrated Care Pathways for the Oral Health Care of People with Learning Disabilities. 2012. http://www.wales.nhs.uk/documents/BSDH_Clinical_Guidelines_PwaLD_2012.pdf. Accessed 26.02 2017.

[32] Public Health England. Local authorities improving oral health: commissioning better oral health for children and young people. An evidence-informed toolkit for local authorities. 2013. https://www.gov.uk/government/uploads/system/uploads/attachment_data/file/321503/CBOHMaindocumentJUNE2014.pdf. Accessed 1.07 2017.

[33] American Academy of Pediatrics. Maintaning and improving the oral health of young children. Pediatrics. 2014;34(6) 10.1542/peds.2014-2984 25422016

[34] American Academy of Paediatrics. Oral health risk assessment timig and establishment of the dental home. Pediatrics. 2003;111(3) 10.1542/peds.111.5.1113 12728101

[35] Pucca G, Riani Costa JF, de Deus Chagas L, Sivestre RM. Oral Health policies in Brazil. Braz oral res. 2009;23(1) 10.1590/s1806-83242009000500003 19838553

[36] Nascimento A, Moysés ST, Werneck RI, Moysés SJ (2013). Oral health in the context of primary care in Brazil. Int Dent J.

[37] Pucca G, Gabriel M, de Araujo ME, de Almeida FCS (2015). Ten years of a National Oral Health Policy in Brazil: innovation, boldness, and numerous challenges. J Dent Res.

[38] Pucca G, Gomes de Lucena EH, Cawahisa PT. Financing national policy on oral health in Brazil in the context of the Unified Health System. Brazilian Oral Research. 2010;24(Spec. Iss 1):26–32. 10.1590/s1806-83242010000500005 20857072

[39] Lima Chaves S (2012). Oral health in Brazil: the challenges for dental health care models. Braz Oral Res.

[40] Arthur T, Rozier RG (2016). Provision of preventive dental services in children enrolled in Medicaid by nondental providers. Paediatrics..

[41] Sams L, Rozier GR, Wilder RS, Quinonez RB. Adoption and implementation of policies to support preventive dentistry initiatives fo rPhysicians: a National Survey of Medicaid programs. Am J Public Health. 2013;103(8) 10.2105/AJPH.2012.301138 PMC4007883 23763420

[42] Hanlon C. Reimbursing Medical providers for preventive oral health services: state policy options. National Academy for state Health Policy 2010. http://nhoralhealth.org/blog/wp-content/uploads/2009/11/PewReimbursingMedicalProviders2.10.pdf. Accessed 22.10 2016.

[43] Damiano P, Reynolds JC, McKernan SC, Mani S, Kuthy R. The need for defining a patient- centered dental home model in the era of the affordable care act. 2015. http://ppc.uiowa.edu/sites/default/files/pchdjul2015.pdf. Accessed 03.26 2017.

[44] Minnesota Department of Human Services. Recomandations for improving oral health services delivery system. Legislative report. 2014. https://www.leg.state.mn.us/docs/2014/mandated/140261.pdf. Accessed 29.06 2017.

[45] Patel R. The State of Oral Health in Europe - Report Commissioned by the Platform for Better Oral Health in Europe. 2012. http://www.oralhealthplatform.eu/wp-content/uploads/2015/09/Report-the-State-of-Oral-Health-in-Europe.pdf. Accessed 1.07 2017.

[46] Heath S. Local authorities’ public health responsibilities. Public Health England. 2014. http://researchbriefings.parliament.uk/ResearchBriefing/Summary/SN06844#fullreport. Accessed 1.07 2017.

[47] NHS commissioning Board. Securing excellence in commissioning NHS dental services. NHS England. 2013. https://www.england.nhs.uk/wp-content/uploads/2013/02/commissioning-dental.pdf. Accessed 28.03 2017.

[48] British Dental Association. Oral Health Inequalities Policy. British Dental Association. 2009. https://www.bda.org/dentists/policy-campaigns/research/government/leg-regs/pub-health-reform/Documents/oral_health_inequalities_policy.pdf. Accessed 15.01 2017.

[49] Haughney M, Devennie JC, Macpherson LM, Mason DK (1998). Integration of primary care dental and medical services: a three-year study. Br Dent J.

[50] Pronych G, Brown EJ, Horsch K, Mercer K (2010). Oral health coordinators in long-term care--a pilot study. Special Care in Dentistry.

[51] Gerritsen P, van der Bilt A, Cune MS, Schrijvers AJ, de Putter C (2013). Integrated versus incidental dental care in nursing homes. Spec Care Dentist.

[52] Dyson K, Kruger E, Tennant M (2012). Networked remote area dental services: a viable, sustainable approach to oral health care in hallanging environments. Aust J Rural Health.

[53] Hom J, Lee JY, Silverma J, Cassamassiomo PS (2013). State Medicaid early and periodic screening, diagnosis and treatment guidelines. JADA.

[54] Kranz A, Rozier RG, Presisser JS, Stearns SC, Weinberger M, Lee JY (2014). Preventive services by medical and dental providers and treatment outcomes. J Dent Res.

[55] Grisanti S, Boyd L, Rainchuso L (2015). An Assessment model for evaluating outcomes in federally qualified health Centers' dental departments: results of a 5 year study. J Dent Hyg.

[56] DiMarco M, Fitzgerald K, Taylor E, Marino D, Huff M, Biordi D, Mundy E (2016). Improving oral health of young children: an interprofessional demonstration project. Pediatr Dent Care.

[57] Bain H, Goldthorpe G. The University of Toronto “Sioux lookout project”-a model of health care delivery. Can Med Assoc J. 1972;107(6) PMC1940911 5057009

[58] Rozier R, Sutton BK, Bawden JW, Haupt K, Slade GD, King RS (2003). Prevention Of early childhood caries in North Carolina medical practices: implications for research and practice. J Dent Educ.

[59] Wysen K, Hennessy PM, Lieberman MI, Garland TE, Johnson SM (2004). Kids Get Care: integrating preventive dental and medical care using a public health case management model. J Dent Educ.

[60] Heuer S. Integrated Medical and Dental Health in Primary Care. JSPN. 2007;12(1). 10.1111/j.1744-6155.2007.00091.x 17233670

[61] Stevens J, Hiroko I, Ingersoll G (2007). Implementing an oral health program in a group prenatal practice. JOGNN.

[62] Pine CM, Dugdill L (2011). Analysis Of a unique global public-private partnership to promote oral health. Int Dent J.

[63] Dugdill L, Pine CM (2011). Evaluation Of international case studies within ‘Live.Learn.Laugh.’: a unique global public-private partnership to promote oral health. Int Dent J.

[64] Brownlee B. Oral health integration in the patient-centered medical home environment: case studies from community health centers. Qualis Health/DentaQuest Foundation. 2012. http://www.qualishealth.org/sites/default/files/white-paper-oral-health-integration-pcmh.pdf. Accessed 1.07 2017.

[65] Maxey H. Integration of oral health with primary care in health centers: profiles of five innovative models. National Association of Community Health Centers. 2015. http://nachc.org/wp-content/uploads/2015/06/Integration-of-Oral-Health-with-Primary-Care-in-Health-Centers.pdf. Accessed 1.07 2017.

[66] Nycz G. The Importance of Medical/Dental Integration in the Care of Diabetes Patients. In: NASHP 27th Annual State Health Policy Conference. Atlanta, Georgia. 2014. https://nashp.org/case-study-bridging-medical-dental-care-marshfield-clinic-family-health-center/. Accessed 6 Feb 2018.

[67] Taflinger K, West E, Sunderhaus J, Hilton I (2014). Health Partners Of western Ohio integrated care case study. CDA Journal..

[68] Gesko D (2014). Health partners: integrated care case study. CDA Journal..

[69] Ramos Gomez F (2014). A model for community-based pediatric oral heath: implementation of an infant oral care program. International Journal of Dentistry.

[70] Partners L. Dental care in accountable care organizations: insights from 5 case. studies. 2015; http://www.ada.org/~/media/ADA/Science%20and%20Research/HPI/Files/HPIBrief_0615_1.pdf?la=en. Accessed 1.07 2017

[71] Acharya A. Marshfield Clinic Health system: integrated care case study. CDA Journal. 2016;44(3) 27044239

[72] Langelier M. The integration of oral health with primary care services and the use of innovative oral health workforce in Federally Qualified Health Centers. In: American Association of Medical Colleges Health Workforce Research Conference. Alexandria, Virginia. 2015. http://www.oralhealthworkforce.org/wp-content/uploads/2017/01/042015a.pdf. Accessed 15.04 2017.

[73] Wooley S (2016). Nganampa health council Denatl program: remote dentistry in the Australian Desert - partnership or perish. Journal of Health Care for the Poor and Undeserved.

[74] Institute of Medecine and National Research Council (2015). Settings in oral health. Improving access to oral health care for vulnerable and underserved populations.

[75] United Nations. Sustainable developmental goals. United Nations. http://www.un.org/sustainabledevelopment/. Accessed 30.06 2017.

[76] World Health Organisation. Integrated care models: an overview 2016. http://www.euro.who.int/__data/assets/pdf_file/0005/322475/Integrated-care-models-overview.pdf. Accessed 1.07 2017.

[77] Lewis R, Rosen R, Goodwin N, Dixon J. Where next for integrated care organisations in the English NHS? 2010. https://www.nuffieldtrust.org.uk/files/2017-01/where-next-integrated-care-english-nhs-web-final.pdf. Accessed 13.12 2016.

[78] Curry M, Ham C. Clinical and service integration. King's Fund. 2010. https://www.kingsfund.org.uk/sites/default/files/Clinical-and-service-integration-Natasha-Curry-Chris-Ham-22-November-2010.pdf. Accessed 6 Feb 2018.

[79] Nolte E, Knai C, McKee M. Managing chronic conditions - Experience in eight countries. European Observatory of Health Systems and Policies. 2008. http://www.euro.who.int/__data/assets/pdf_file/0008/98414/E92058.pdf. Accessed 15.03 2017.

[80] Merzel C, D’Afflitti J (2003). Reconsidering community-based health promotion: promise, performance, and potential. Am J Public Health.

[81] Silva de Souza T, Roncalli AG (2007). Oral health in the Brazilian family health program: a health care model evaluation. Cad Saúde Pública.

[82] Guerra Aquilante A, Gurgel Aciole G (2015). Building a “smiling Brazil”? Implementation of the Brazilian National Oral Health Policy in a health region in the state of São Paulo. Cad Saúde Pública..

[83] Arksey H, Scoping O'ML (2005). Studies: towards a methodological framework. Int J Soc Res Methodol.

